# Vehicle Counting and Moving Direction Identification Based on Small-Aperture Microphone Array

**DOI:** 10.3390/s17051089

**Published:** 2017-05-10

**Authors:** Xingshui Zu, Shaojie Zhang, Feng Guo, Qin Zhao, Xin Zhang, Xing You, Huawei Liu, Baoqing Li, Xiaobing Yuan

**Affiliations:** 1 Science and Technology on Microsystem Laboratory, Shanghai Institute of Microsystem and Information Technology, Chinese Academy of Sciences, Shanghai 201800, China; zuxs@mail.sim.ac.cn (X.Z.); GuoFeng@mail.sim.ac.cn (F.G.); qinzhao21@mail.sim.ac.cn (Q.Z.); livia138567@126.com (X.Y.); liuhw@mail.sim.ac.cn (H.L.); sinowsn@mail.sim.ac.cn (X.Y.); 2 University of Chinese Academy of Sciences, Beijing 100049, China; 3 School of Mechanical Engineering and Electronic Information, China University of Geosciences, Wuhan 430074, China; 20141001117@cug.edu.cn; 4 IBM-Research China Labotatory, Beijing 100094, China; zxinscholar@gmail.com

**Keywords:** vehicle counting, moving direction, small-aperture microphone array, UGS

## Abstract

The varying trend of a moving vehicle’s angles provides much important intelligence for an unattended ground sensor (UGS) monitoring system. The present study investigates the capabilities of a small-aperture microphone array (SAMA) based system to identify the number and moving direction of vehicles travelling on a previously established route. In this paper, a SAMA-based acoustic monitoring system, including the system hardware architecture and algorithm mechanism, is designed as a single node sensor for the application of UGS. The algorithm is built on the varying trend of a vehicle’s bearing angles around the closest point of approach (CPA). We demonstrate the effectiveness of our proposed method with our designed SAMA-based monitoring system in various experimental sites. The experimental results in harsh conditions validate the usefulness of our proposed UGS monitoring system.

## 1. Introduction

The motion parameters of vehicles are important intelligence for a unattended ground sensor (UGS) system and Intelligent Transport System (ITS) [1,2,3,4,5,6]. The fixed-point observation technique is widely employed in UGS and ITS by installing an inductive loop sensor [7], ultrasonic sensor [8,9], seismic sensor [10] and camera [11]. However, these sensing systems suffer from complicated installation, expensive maintenance costs and high power consumption.

The microphone array (MA) sensor provides low cost, low power consumption, and non-line-of-sight measurement, which is widely used for acquiring military intelligence of intruding targets [1,3,6,12]. A network of MA-based surveillance sensors remotely deployed in conjunction with a command center can provide early warning and assessment of enemy threats, near real-time situational awareness to commanders, and may reduce potential hazards to soldiers [13]. Furthermore, the employment of small-aperture microphone array (SAMA) as a monitoring sensor should be an undetectable system which can avoid visual problems and be cheaper than most currently used systems.

The MA-based detection and classification of moving targets in UGS have received much attention [14,15,16,17,18]. However, relatively less attention has been paid to the counting and moving direction estimation in UGS. This issue generally relates to the detection of moving targets, and more specifically to the identification of the number and moving direction of vehicles using one or more SAMA sensors. More particularly, this issue pertains to a system that uses acoustic sensors to acquire enough intelligence on moving targets in dynamic, noisy, and highly mobile environments. An ’acoustic trip line’ counter was introduced in [6,13] to detect the harmonic components in the prescribed look-direction by MA with the radius of 0.612 m. Moreover, the method of collaborative signal processing of a pair of 1 m spacing MA sensors was designed in [19] to determine the number of targets. Nevertheless, those currently used large aperture systems destroyed the stealth and installability characteristic of acoustic sensor in UGS. On the other hand, traffic monitoring systems with MA applied in ITS are proposed in [1,2,3,20]. However, both of the methods proposed in [1,2] need channel synchronization and ultra-high sampling frequency (48 kHz), which are not realistic in a UGS system. Furthermore, a system based on the amplitude or energy of vehicle-generated sound is easily spoofed or counter measured in military application.

The counting of vehicles and the estimation of their moving direction with small aperture and lower sampling rate are a knotty problem. Another challenge for the SAMA sensor in the wild environment is the wind-generated noise. The sound of the vehicle in the real-world environment has free-field characteristics, and the wind noise has noise-field characteristics [21]. It is well known that the wind noise is unavoidable since it cannot be totally removed by a wind shield. According to [21], spatial coherence could be used to distinguish between the noise of the wind and the sound of the vehicle for each frequency bin. Therefore, we employed a spatial coherence-based method to select the useful bands for determining the vehicle direction, counting vehicles and estimating their moving direction.

In this paper, a SAMA-based system for counting vehicles and estimating their moving direction is provided and its aperture is only 4 cm. We defined a decision zone (DZ) near to the closest point of approach (CPA) to identify the number and moving direction of vehicles. Since the direction of arrival (DOA) estimation error around the CPA is relatively small, we can achieve higher estimation accuracy. The interference of wind noise in the real-world environment is reduced through the estimation of the useful frequency bands by spatial coherence.

This paper is organized as follows. Section 2 illustrates the design of the SAMA sensor system, including the system hardware architecture and the DOA estimation algorithm. Section 3 describes the vehicle counting and moving direction estimation scheme based on the calculated DOA. System verification and experimental results in different situations are given in Section 4 and conclusions are presented in Section 5.

## 2. Design of the Estimation System

### 2.1. SAMA System Architecture Design

In general, the uniform array can provide balanced space for circuit design and the uniform circular array (UCA) has the same resolution in all directions. The vehicle signal occupies the frequency bands from 100 Hz to 3000 Hz [22]. The aperture of the array has to satisfy the spatial sampling criterion in all the frequency bands to avoid performance degradation due to spatial aliasing. Therefore, to satisfy the spatial sampling criterion d≤0.5λ, the array aperture should be no bigger than 5 cm, where d is the minimum distance between any two array microphones, and λ is the wavelength of the acoustic signal. Finally, uniform circular geometry with an aperture of 4 cm is employed to deploy the microphone [23].

**Figure 1:**
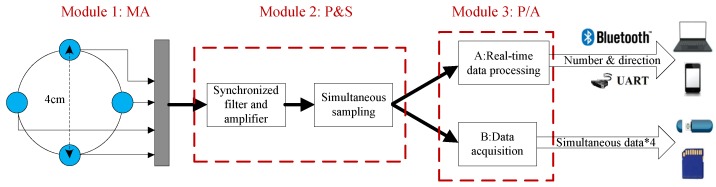
Block diagram of the small-aperture microphone array (SAMA) system hardware architecture.

The block diagram of the prototype SAMA system is depicted in Figure 1. The system is divided into three modules according to their functions: MA module (Module 1: MA); preprocessing and sampling module (Module 2: P&S); and real-time processing or data acquisition module (Module 3: P/A). The acoustic signals from the MA module are sampled in the P&S module to obtain four simultaneous digital signals. The synchronized filters and amplifiers mean that a comparatively strict demand on the consistency of the four channels is requested. The function of the P/A module is configured by users, either for real-time processing by digital signal processing (DSP) or for storing the signals in the memory device for further analysis.

**Figure 2:**
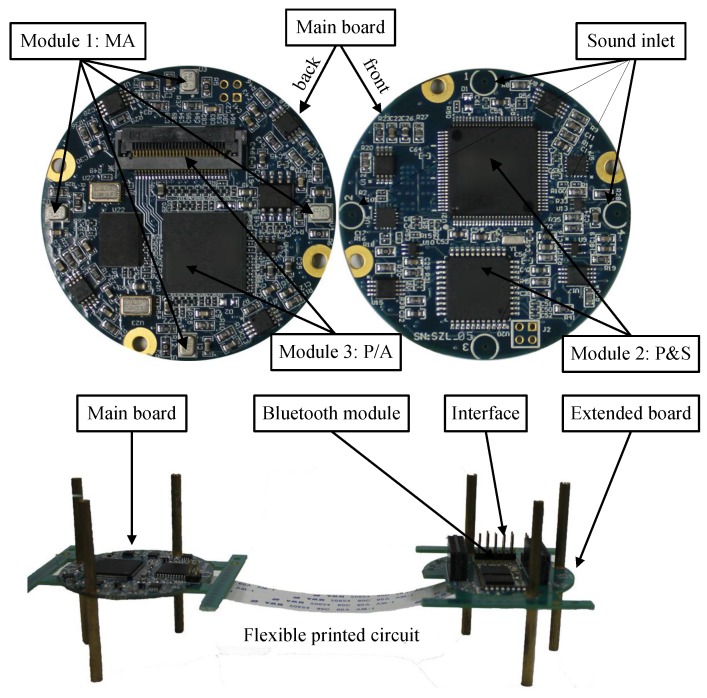
Photograph of the SAMA system; array aperture is 4 cm.

As shown in Figure 2, the system consists of a mainboard as well as an extended board connecting by a flexible printed circuit. The mainboard consists of a UCA system with four ADMP504 MEMS microphones (Analog Devices, Norwood, MA, USA), a DSP (ADSP21375, Analog Devices, Norwood, MA, USA) as the core processor, MAXIM MAX11043, four-Channel, 16-Bit analog-to-digital converters (ADCs) (Maxim Integrated Products, Sunnyvale, CA, USA) and supplemental hardware circuits. The MAX11043 contains one versatile filter block and programmable-gain amplifier per channel. The extended board contains a CSR BC6415 Bluetooth module (Cambridge Silicon Radio, Cambridge, UK), a data acquisition interface and debug interface. The hardware components that make up the system are illustrated in Figure 2. In general, the aperture of our system is very small (4 cm) which is an advantage for portability and mobility, but a challenge for high accuracy DOA estimation [23].

### 2.2. DOA Estimation with Spatial Coherence

DOA estimation using acoustic signals is inevitably contaminated by wind noise which is the most common interference in an outdoors environment. The wind turbulence on the microphone is comparatively incoherent, and its speed is much slower than that of sound [24]. Spatial coherence is a similarity indicator for signals in the frequency domain. It describes the coherence between two measures at two locations [21]. The spatial coherence function between two microphone signals, $x_{1}$ and $x_{2}$, is equal to the cross power spectrum $G_{x_{1}x_{2}}\left(f\right)$ divided by the square root of the product of the two auto-power spectra. Specifically, the spatial coherence of $x_{1}$ and $x_{2}$ is defined by Equation (1):

(1) $$\gamma_{x_{1}x_{2}}\left(f\right)=\frac{G_{x_{1}x_{2}}\left(f\right)}{\sqrt{G_{x_{1}x_{1}}\left(f\right)G_{x_{2}x_{2}}\left(f\right)}},$$

where *f* denotes the frequency of interest. The complex cross power spectrum defined in Equation (2) is the Fourier transform of the cross correlation of $x_{1}$ and $x_{2}$ in Equation (3).

(2) $$G_{x_{1}x_{2}}\left(f\right)=\int_{-\infty }^{\infty }R_{x_{1}x_{2}}\left(\tau \right)e^{j2\pi f\tau }d\tau ,$$

(3) $$R_{x_{1}x_{2}}\left(\tau \right)=E\left[x_{1}\left(t\right)\;x_{2}\left(t+\tau \right)\right].$$

Here, $x_{1}$ and $x_{2}$ are two different channel signals from SAMA and *E* denotes the mathematical expectation (for ergodic random processes the ensemble average can be replaced by a time average). Carter [25] gives an analytical estimation of the bias $E\left[{\left|{\hat{\gamma }}_{x_{1}x_{2}}\right|}^{2}\right]-{\left|{\hat{\gamma }}_{x_{1}x_{2}}\right|}^{2}$ as a function of the true spatial coherence ${\left|{\hat{\gamma }}_{x_{1}x_{2}}\right|}^{2}$, the fast fourier transformation (FFT) time duration *T* and the time delay *D*.

(4) $$E\left[{\left|{\hat{\gamma }}_{x_{1}x_{2}}\right|}^{2}\right]-{\left|{\hat{\gamma }}_{x_{1}x_{2}}\right|}^{2}≅\frac{-2\left|D\right|}{T}{\left|{\hat{\gamma }}_{x_{1}x_{2}}\right|}^{2}+{\left(\frac{\left|D\right|}{T}\right)}^{2}{\left|{\hat{\gamma }}_{x_{1}x_{2}}\right|}^{2}.$$

**Figure 3:**
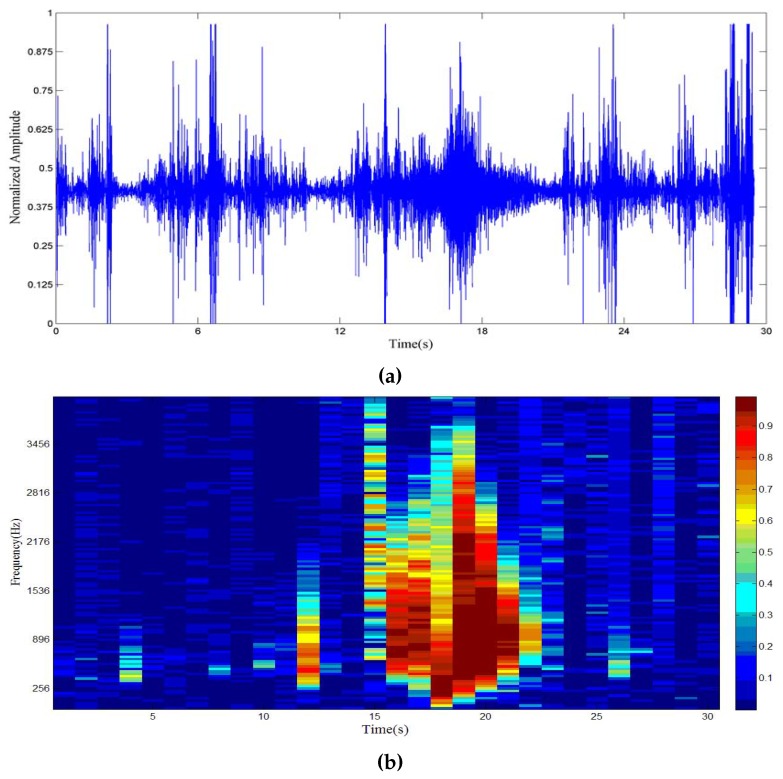
(**a**) Acoustic signal of a car passing the SAMA in an outfield test; (**b**) Spatial coherence of (**a**).

In our case, *T* = 125 ms (1024 sampling points), *D* = 8.31 × 10^−5^ s (array aperture of 4 cm). Figure 3a shows the acoustic signal of a car passing the SAMA sensor and the wind scale [26] is 4. Spatial coherence is depicted in Figure 3b to show whether the frequency bin is dominated by vehicle or wind noise. To identify the useful frequency band of the signal, we check whether the spatial coherence is above the threshold in each frequency bin. In this paper, 0.7 is chosen by simulation and experiment. If the spatial coherence of a certain frequency bin is larger than 0.7, then this bin will be selected for direction finding and other uses; otherwise, it will be discarded.

An improved multiple signal classification (MUSIC) algorithm is employed to DOA estimation associated with spatial coherence to discriminate between the wind noise and the acoustic signal of a vehicle. The algorithm first tests the spatial coherence for each frequency bin, then identifies the useful frequency bands for wind noise robust DOA estimation. Details of identifying spatial coherence and selecting useful frequency bands for DOA estimation are discussed in [23].

In addition, inspired by [2], we designed an inaccurate angle regulation (IAR) method to adjust the estimated target angle. One angle value calculation is performed on a signal length of 125 ms (frame length) and the frame moving step is equal to the frame length. If motion speed is 60 km/h, the vehicle moves about 2 m at this time interval, which corresponds to the angle deviation of 0°–11° (assuming that sensors are displaced 10 m away from the road). This angle deviation depends upon the location of the vehicle in relation to the MA. We conclude that it is not the correct angle of the vehicle and that maybe it is falsely estimated if the two closest neighbor angle values differ more than a deviation of 11° (deviation threshold). Then, the neighboring angles will linear fitting out a value to replace it. Following the aforementioned principle, the deviation threshold is proportional to the speed of the vehicle.

## 3. Vehicle Counting and Moving Direction Estimation

**Figure 4:**
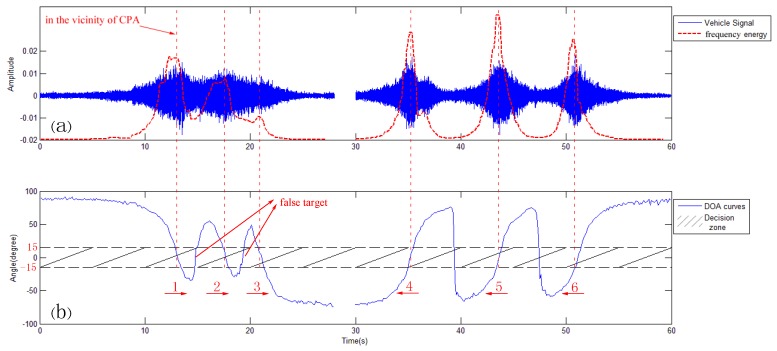
(**a**) Six vehicles passing through SAMA sensors within 1 min; (**b**) the corresponding direction of arrival (DOA) curves of (**a**).

**Figure 5:**
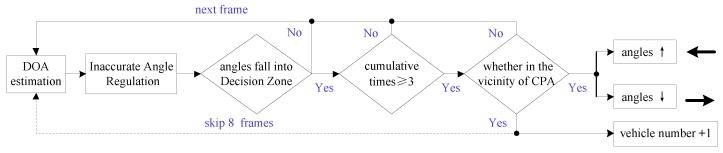
The flowchart of the algorithm for vehicle counting and moving direction estimation.

**Figure 6:**
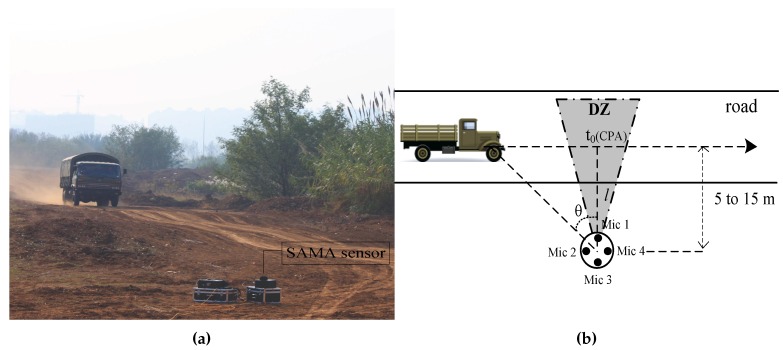
(**a**) Photograph of the recording setup; (**b**) Illustration of the DOA estimation scenario.

In this section, we describe the number and moving direction estimation method of vehicles travelling on a previously established route with the calculated DOA. Through statistical analysis, the varying trend of vehicle’s bearing angles can provide the number information of vehicles, as shown in Figure 4a. Since the angle estimation error around the CPA is relatively small, we defined a DZ $\left(-15^{{}^{\circ}},+15^{{}^{\circ}}\right)$ around the CPA $\left(0^{{}^{\circ}}\right)$ for subsequent processing. Then, we designed a vehicle counting and moving direction estimation method by analyzing the relationship of the angles with the DZ as described in Figure 5. The reference coordinate system is shown in Figure 6b.

Angles that fall into the DZ more than or equal to three times represent a vehicle that was detected passing through a SAMA sensor. Subsequently, the number of vehicles is increased by 1 and the moving direction can be obtained by checking the varying trend of the angles. The vehicle is approaching from the left direction if the angles are gradually decreasing, and conversely from the right direction. At this stage, the detection of a vehicle and its moving direction is finished. After those operations, we skip eight frames to avoid the repeated count of the same vehicle because, in such a short time, another vehicle will not appear.

**Figure 7:**
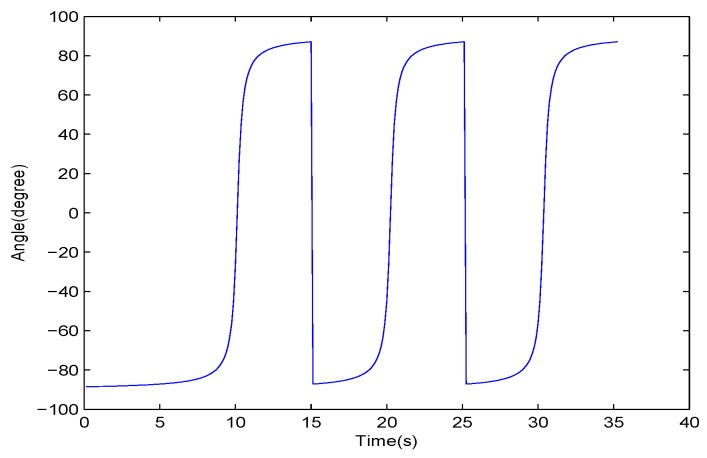
The simulated ideal DOA curve of three vehicles passing a SAMA sensor.

Assuming that the velocity of the vehicle (denoted as *v*) is uniform, then its DOA satisfies the inverse tangent law as Equation (5):

(5) $$\theta =\arctan\frac{v\cdot (t-t_{0})}{l},t\in R,$$

where $t_{0}$ represents the moment of CPA and *l* represents the distance between the sensors and lane center, as depicted in Figure 6. Moreover, the ideal DOA curve of three vehicles, without them mutually interference with each other, is shown in Figure 7. However, due to the mutual interference of vehicles, the actual DOA curve of three vehicles passing a SAMA sensor is shown as 0 s to 30 s in Figure 4b. Hence, our method will cause false targets in the middle of two vehicles, as annotated in Figure 4b. As the DZ is very close to $0^{{}^{\circ}}$, the vehicle is close to the CPA. Therefore, as shown by the red dotted line in Figure 4a, the frequency energy of selected frequency bands in Section 2.2 is employed to roughly judge whether those frames are in the vicinity of the CPA. Then, we can exclude false targets in order to achieve a highly accurate vehicle count.

Since counting and moving direction estimation are based on the same vehicle, we should not analyze either of them individually. The estimation of moving direction is executed immediately once a vehicle is counted.

DOA estimation is easily disturbed by other interference signals when the target is far away from the CPA, and the DOA estimation error is positively related to the distance from the CPA. The proposed method, based on the DZ, tightly surrounds the CPA which makes the method more effective.

## 4. System Verification and Experimental Results

### 4.1. Experimental Conditions and Datasets

Ground vehicles are moving targets of focal interest to UGS. Therefore, these three types of vehicles are employed in our experiment and part of their specifications is listed in Table 1. The acoustic signal of a moving target is sampled by the SAMA sensor with a 8192 Hz sampling rate. Besides, each vehicle is equipped with GPS to obtain the velocity and distance information between the moving target and the sensor. The sensor was located parallel to the road, about 5 to 15 m away from the lane center and the experimental layout is shown in Figure 6. Meanwhile, wind scales were recorded by an ultrasonic anemometer at the same site during our outfield experiments.

**Figure 8:**
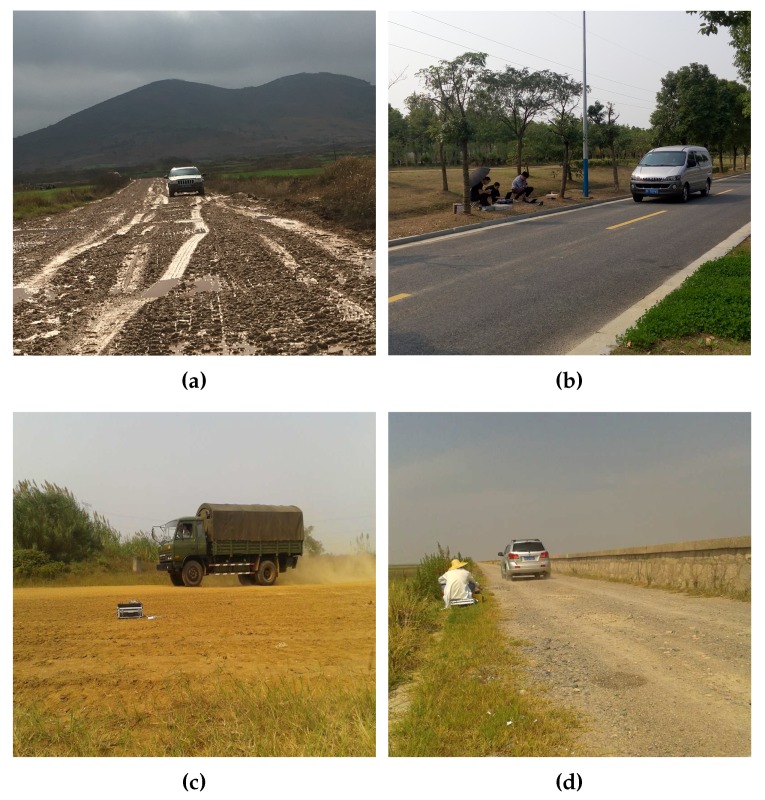
Four different experimental environments in Nanjing, Anhui and Shanghai. (**a**) dirt road; (**b**) concrete road; (**c**) mud road; (**d**) sand road.

The noise emitted by a vehicle at low and medium speeds is composed of tyre/road noise and mechanically originated noise [27]. Considering the effect of road type and speed, experiments were conducted in different terrains at different speeds [28]. Experimental studies were performed from June 2013 to December 2016 on Chongming Island, Zhoushan Island, Nanjing, Anhui and a suburban district around Shanghai where the wind scales are usually less than 6. The compositions of our sample set are shown in Table 2 and the photographs of four experimental environments are shown in Figure 8. In those experimental sites, vehicles move at the predetermined velocity of 30 to 60 km/h when datasets are collected. Some of the measurement sites are military training grounds with some background activity at times. Samples with higher speeds are not available for the bad road conditions in the real-world environment. Moreover, for the sake of security, experiments of tracked vehicles driving at 60 km/h on the concrete road were not implemented.

### 4.2. Results and Discussion

**Figure 9:**
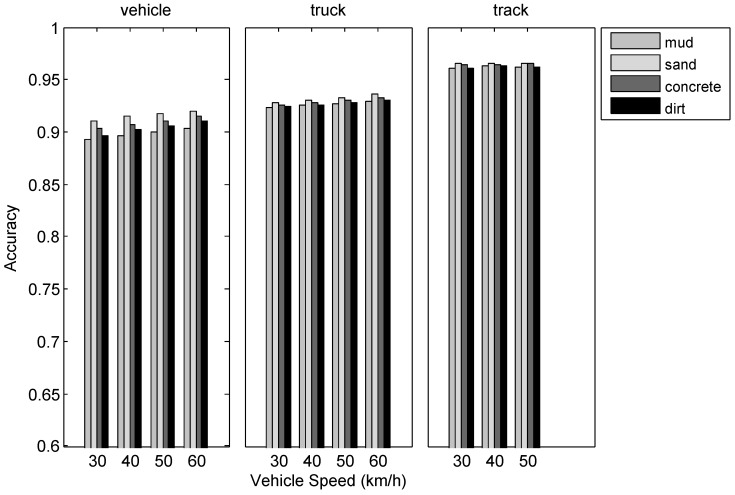
Evaluation results of the vehicle counting algorithm using collected datasets.

In this section, the experimental data are cropped and a total of 306 min useful acoustic samples from different sites are analyzed. Figure 9 presents the counting accuracy for three types of vehicles in four terrains with different speeds. According to Figure 9, the influence of road type to counting accuracy is greater than that of vehicle speed. In addition, these two factors have the greatest impact on the counting accuracy of cars, while they have less and the least impact on trucks and tracked vehicles, respectively. The faster the speed, the greater the vehicle noise, thus higher counting accuracy can be achieved. Moreover, regardless of the type of vehicle, the counting accuracy on the sand road is the highest among all the tested terrains.

The average counting accuracy of three types of vehicles is shown in Table 3 without considering the effect of the terrain and speed. The accuracy of the tracked vehicle is as high as 96.42% due to its high level of noise. The special counting mechanism ensures that it will not lead to false positive detection, even in strong wind weather conditions. However, ultra-close-distance (less than 20 m) driving and overtaking are the main factors that result in false detection. Fortunately, both aforementioned situations, commonly encountered in ITS, are rare in the process of troops moving. Therefore, the proposed method is suitable for monitoring military activity with UGS.

Since counting and moving direction estimation are based on the same vehicle, a one-to-one relationship exists between them. Through our statistics, the moving directions of all correctly counted vehicles are correctly estimated (accuracy: 100%). It is worth emphasizing that the falsely counted vehicles are not involved in the calculation. Considering the decision mechanism, judging the increasing or decreasing trend of angles in the vicinity of the CPA, the result is reliable. Because of the 100% estimation accuracy of the moving direction, the overall performance of the system is dominated by counting accuracy.

Considering the application in the field environment, in contrast to ITS, vehicle speed is slow and vehicle flow is limited. Therefore, we can achieve satisfactory results of vehicle counting and moving direction estimation with small aperture and lower sampling rate. However, the ITS has the characteristics of faster vehicle speed, traffic intensity and multi-lane interference. Consequently, it needs large aperture arrays, synchronous acquisition and a very high sampling rate. Those demands lead to a high power consumption and make the UGS system difficult to implement. Hence, we did not compare the performance of the proposed method with methods in [1,2]. Certainly, we should admit that our proposed method shows poor ability in some of the ITS application scenarios.

The proposed method realizes the counting and moving direction estimation issue through the proposed algorithm with only 4 cm aperture array. The method also introduces spatial coherence for wind noise suppression and IAR to overcome the interference from unrelated targets. In the situation of different types of closely spaced vehicles, however, the method failed because the acoustic signals are dominated by very noisy vehicles. Then, the problem of separating signals in multiple target scenarios needs to be solved, which will be considered in future work.

## 5. Conclusions

In this paper, we proposed a SAMA-based single node acoustic monitoring system with only 4 cm aperture. The proposed method includes vehicle counting and motion direction estimation. The method obtains the required intelligence by analysing the varying trend of a moving vehicle’s angles within the vicinity of the CPA. Spatial coherence was assessed to select frequency bands for wind noise suppression and DOA estimation. We applied our proposed system to four different experimental environments, and assessed the accuracy of vehicle counting and motion direction estimation. The experimental results in harsh conditions confirmed the availability of our proposed UGS monitoring system.

## Figures and Tables

**Table 1:** Different vehicles’ specifications.

	Vehicle Types		
	Car	Truck	Tracked Vehicle
Weight (kg)	1425	6800	40,200
Number of cylinders	4	6	10
Engine capacity	78	170	3240
Samples (min)	107	104	95

**Table 2:** Experimental datasets recorded in four different experimental sites; every sample is 60 s with a sampling rate of 8192 Hz.

Recording Location		Chongming	Zhoushan	Fengxian	Nanjing
**Road Type**		**Dirt**	**Concrete**	**Sand**	**Mud**
Vehicle Type	Car	24	30	25	28
	Truck	26	28	22	28
	Tracked	31	0	30	34
Total Samples (min)		81	58	77	90

**Table 3:** The average accuracy of the counting algorithm, ignoring the effect of the terrain and speeds.

Vehicle Type	Car	Truck	Tracked Vehicle
Average Accuracy	90.67%	92.86%	96.42%
